# Supplementary figures and images for: Estimating the one-repetition maximum on the leg-press exercise in female breast cancer survivors
Source: PeerJ. 2023 Sep 27;11:e16175. doi: 10.7717/peerj.16175 (PMC10541811; doi:10.7717/peerj.16175)

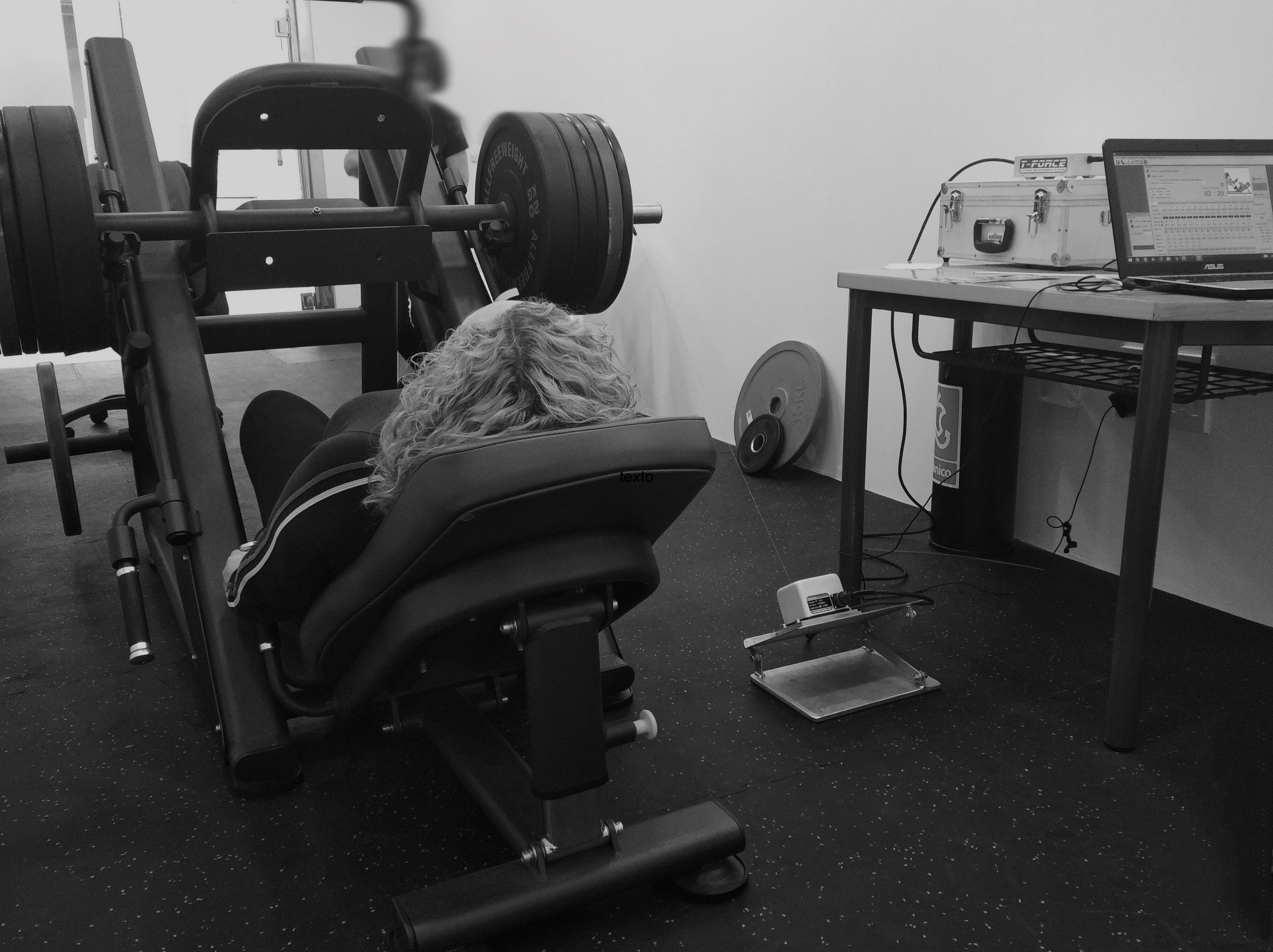

Supplement: Supplemental Information 2 [file peerj-11-16175-s002.jpg]
